# The Effects of Locus Coeruleus and Norepinephrine in Methamphetamine Toxicity

**DOI:** 10.2174/157015913804999522

**Published:** 2013-01

**Authors:** Michela Ferrucci, Filippo S Giorgi, Alessia Bartalucci, Carla L Busceti, Francesco Fornai

**Affiliations:** 1 Department of Human Morphology and Applied Biology, University of Pisa, Pisa, Italy; 2 Department of Neurosciences, section of Neurology, University of Pisa, Pisa, Italy; 3 Laboratory of Neuroanatomy of Movement Disorders, I.R.C.C.S. I.N.M. Neuromed, Pozzilli (IS), Italy

**Keywords:** Behaviour, Dopamine, Locus Coeruleus, Methamphetamine, Neurochemistry, Norepinephrine, Substantia Nigra, Drugs of Abuse.

## Abstract

The activity of locus coeruleus (LC) neurons has been extensively investigated in a variety of behavioural states. In fact this norepinephrine (NE)-containing nucleus modulates many physiological and pathological conditions including the sleep-waking cycle, movement disorders, mood alterations, convulsive seizures, and the effects of drugs such as psychostimulants and opioids. This review focuses on the modulation exerted by central NE pathways on the behavioural and neurotoxic effects produced by the psychostimulant methamphetamine, essentially the modulation of the activity of mesencephalic dopamine (DA) neurons. In fact, although NE in itself mediates some behavioural effects induced by methamphetamine, NE modulation of DA release is pivotal for methamphetamine-induced behavioural states and neurotoxicity. These interactions are discussed on the basis of the state of the art of the functional neuroanatomy of central NE- and DA systems. Emphasis is given to those brain sites possessing a remarkable overlapping of both neurotransmitters.

## 1 THE CHEMICAL NEUROANATOMY OF NE-CONTAINING LC NEURONS

The main source of NE in the brain is represented by an NE pontine nucleus, which corresponds to the nucleus Locus Coeruleus (LC, A6, according to the original catecholamine nuclei classification by Dahlstrom and Fuxe [1]). This is located in the upper part of the floor of the fourth ventricle. This nucleus is highly preserved phylogenically [2-4]. Rather than being a single brain nucleus, LC represents a nuclear complex, which is located bilaterally within the lateral pontine central grey, and includes scattered catecholamine neurons close to the brachium conjunctivum, and within the central tegmental regions of the pontine gray matter [5, 6].

Caudal NE neurons are separated from the LC complex and are located in the medullary ventrolateral reticular formation (A1 and A2 areas, according to the description by Dahlstrom and Fuxe, [1]). They send their axons to restricted target areas and play an important role in regulating autonomic functions and neuroendocrine control [7].

The LC complex is shaped like a tube, and its major cell types are medium-sized neurons, bearing coarse particles of melanin granules to the cytoplasm [8]. In young adult human the LC complex contains approximately 60,000 neurons, which decrease down to 40,000 during normal ageing [9, 10]. Most NE neurons are scattered in the LC complex, while one third are packed within the main pontine LC nucleus [11]. In keeping with neuronal density, the dorsomedial compartment of LC contains densely packed neurons, while in the ventrolateral compartment neurons are dispersed [5].

In humans, the rostrocaudal extension of the LC nuclear complex is approximately 16 mm [3, 12]. The rostral end of LC reaches decussation of the trochlear nerve up to the periacqueductal gray of the mesencephalon, while the caudal end descends to the level of the facial nerve [5]. 

The cytology of LC neurons features both medium- and small-sized neurons, 35-45 µm and 15-25 µm diameter respectively. The medium-sized neurons are multipolar cells generally round in shape, with only a few dendrites located in the rostral part of the nucleus and often extending into surrounding structures. The small spindly LC neurons have two tufts of dendrites and are found within the ventral LC complex [5].

LC axons branch profusely, thus allowing a single NE axon to send terminals throughout the entire cerebral cortex. Nearly all levels of the CNS are thus innervated by LC axons, which provide the sole NE innervation of the isocortex, allocortex, and cerebellar cortex [5, 6, 13-17], while they caudally innervate the medulla, and spinal cord [3, 18].

The multiple divergence of axon collaterals is concomitant with the occurrence of diffuse non synaptic effects on neighbouring cells. This leads to a widespread release of NE from the LC axons which may reach a variety of targets. NE thus acts as a neuromodulator within a circumscribed microenvironment, rather than as a classic neurotransmitter [19, 20]. In fact, NE activity extends beyond neurons and involves astrocytes and cerebral blood vessels. In line with this, NE system also plays a fundamental role in the physiology of the blood-brain barrier and in glial cells activity [21, 22]. This includes NE effects on the microglial release of specific cytokines [23, 24]. The paracrine effects of the central NE system due to the marked divergence of axon collaterals is further sustained by the fine morphology of these nerve endings. In fact, NE axon collaterals possess “boutons en passage”, consisting of varicosities, rather than classic “boutons terminaux” that are typical of non-monoamine axon terminals. The spikes travelling along the fibre are thus able to release NE from multiple serial sites rather than just from the fibre end. 

Interestingly, varicosities along LC axons differ from those described along NE axons arising from other NE nuclei. In fact, while NE axons that arise from LC possess small (0.5 µm) beaded varicosities, axons arising from the medullary A1 and A2 NE cell groups have varicosities with a larger diameter (1±3 µm).

Such a fine anatomical discrepancy has important pathological consequences. In fact, monoamine axons with smaller beaded varicosities have a lower threshold to various neurotoxic insults and are more prone to neurodegeneration compared with the ones bearing large varicosities [25-27]. These considerations call for more in-depth studies aimed at relating the cell biology of synaptic varicosities with selective neuronal vulnerability occurring during neurotoxic insults and neurodegenerative disorders.

## 2 ANATOMICAL CONNECTIONS OF LC NE NEURONS (FIG. 1)

The classic histofluorescence method of Falck* et al., *[28] led to the identification of two main ascending pathways from the LC. These were confirmed by immunohistochemical methods and consist of: 1) a dorsal pathway [29], innervating the entire cerebral cortex, especially motor and premotor areas, the olfactory tubercle, the septum, the bed nucleus of the stria terminalis, the hippocampal formation, and the amygdala [30, 31]; and 2) a ventral or intermediate pathway innervating the hypothalamus, overlapping with NE projections coming from the A1 and A2 regions. Groups of fibers have been described that project from the LC to the subthalamic nucleus [32], substantia nigra (SN, A9 according to Dahlstrom and Fuxe, [1]) [33], and ventral tegmental area (VTA, A10) [34]. Finally, other afferent fibers pass *via *the superior cerebellar peduncle to the cerebellum [35], and a caudal projection has been traced to the reticular formation and the cord as well [36]. The striatum just has a small amount of NE, receiving only scattered fibers from the LC. Nonetheless, these striatal afferents seem to possess a high turnover rate [25, 26, 37]. 

The main afferents to the LC include projections from the prefrontal cortex (PFC), lateral hypothalamus [38], cerebellum [39], raphe nuclei [40], and amygdala [41]. Furthermore, the LC receives NE afferents from lower medullary A1 and A2 regions [42-44]. In addition, LC receives DA afferents from VTA [45]. In line with the specific aim of the review, below is a brief summary on specific sites for NE-DA interaction.

Morphological analysis indicates that the ventral striatum receives direct NE innervation from the LC at the level of the caudal part of nucleus accumbens (NAc) shell [46, 47]. These NE axons also originate from A2 [48]. On the other hand, the rostral part of the NAc as well as the dorsal striatum have scattered NE innervations [46-48]. These data were recently confirmed by direct catecholamine assay, in situ voltammetry, stimulations of the median forebrain bundle and various pharmacological manipulations [49]. The origin of these fibers is only partly attributed to LC, although this issue remains under debate [25].

## 3 THE ANATOMY OF NE-DA INTERACTIONS

The interactions between NE and DA have been subject to intense investigation in the neuroanatomy of catecholamine systems, which appear to be reciprocally connected. In fact, LC receives DA afferents from VTA [45] and there is strong evidence for the presence of detectable NE levels in the VTA which derives from multiple sources [50-52]. In fact, two decades ago it was shown that stimulation of VTA neurons increases the activity of the LC [45]. Similarly, A1, and A2 NE nuclei were shown to provide excitatory stimulation to VTA DA neurons [53, 54]. Further indirect evidence for NE-DA interaction at the level of the VTA has been provided by autoradiographic and immunohistochemical studies, showing alpha1 and alpha2- adrenergic receptor (AR) expression in most DA neurons of the VTA [55, 56]. 

However, the precise source of NE terminals in the VTA was only recently defined in detail [57]. The authors of [58] described the sources of NE in the VTA, SN and retrorubral field (A8). By using retrograde tracing, they showed that NE afferents to the VTA originate mainly from the LC and, to a lesser extent, from A5 (NE neurons placed in the ventrolateral pons); each LC innervates both homo- and contra-lateral VTAs, to a similar extent. Liprando* et al., *[58] described the interaction between NE terminals and VTA DA neurons by using an immune-electron microscopy dual labeling approach both in rats and monkeys. They showed that the vast majority (more than 70%) of interactions between NE transporter (NET)-positive (i.e. NE-containing) terminals and DA neurons occur between NE varicosities and DA dendrites showing ultrastructural features typical of extra-synaptic sites. This thus confirmed once again that NE fibers have a neuromodulatory extra-synaptic effect on their targets [58].

Among mesocortical DA target areas, PFC is densely innervated by LC-originating NE terminals. Knowledge of the functional effects of this innervation added further details to the “functional anatomy” of LC-PFC interactions. For instance, Hertel* et al., *[59] showed that alpha2 AR antagonists increase basal DA output in the medial prefrontal cortex through a direct effect on DA terminals within PFC [59]. 

Concerning the nigrostriatal system, SN receives direct NE projections from LC, in addition to A1 and A2 nuclei [57].

While anatomical evidence remains unclear, the presence of NE in the dorsal striatum is widely established, as well as high NE turnover, as witnessed by the high ratio between 3-methoxy4-hydroxyphenylglycol (a major metabolite of NE) and NE in the striatum itself (see for instance [25, 26, 60]). This might explain why damage to LC decreases striatal DA release [25, 26, 61-65]. The anatomical connections between LC NE neurons and DA areas is critical to understanding the behavioural effects induced by methamphetamine (METH). In fact, the role of DA in mediating the action of METH changes dramatically depending on the activity of the LC-NE system. 

In order to dissect this multi-faceted issue in Section 4 we summarize the molecular events produced by METH on DA and NE containing cells in order to explain the behavioural effects.

## 4 MOLECULAR EFFECTS OF METHAMPHETAMINE (METH) ON CATECHOLAMINE (DA AND NE) CELLS

Methamphetamine acts on multiple classes of neurons as well as different molecular targets. In fact a variety of neurotransmitters are released under METH administration - namely, serotonin [66] glutamate [67-69] and acetylcholine [70, 71]. Among these, catecholamine neurons are considered as the main target since METH is a powerful DA and NE releaser [72, 73]. As a consequence, repetitive administration of METH leads to DA-and NE-dependent behavioural sensitization, which can be reproduced in different species.

**Fig. (2):**
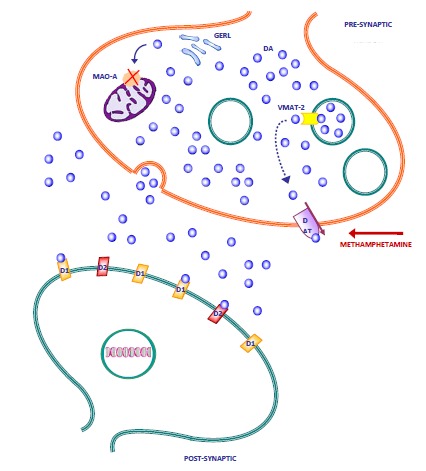
**Neurochemical targets of methamphetamine.** The picture shows the biochemical effects of amphetamine derivatives within a DA nerve terminal: 1) the vesicular monoamine transporter (VMAT) type 2, which is responsible for the storage of DA within the synaptic vesicles; 2) the DA transporter (DAT), which takes up DA from the synaptic cleft to the DA axons; 3) the MAO-A, which is responsible for oxidative deamination of DA. Methamphetamine impairs the activity of these proteins at several levels: 1) by reverting the direction of DA transport of both VMAT-2 and DAT; 2) by myslocating VMAT-2 from synaptic vesicles to other membrane systems (i.e. GERL); 3) by inhibiting the activity of the MAO-A. This results in a massive efflux of DA first into the nerve terminal and then into extracellular space. Free DA autoxidates and produces highly reactive intermediate species which are responsible for the oxidative alteration of several proteins and lipids (for a review see [97]). Similar molecular targets can be identified in NE nerve terminals. Methamphetamine has a 10-fold higher affinity for the NE transporter (NET) than DAT. In fact, the same dose of methamphetamine produces an NE release higher than the DA release [72]. This effect might explain the association between NE release and behavioural effects induced by methamphetamine. The effects produced by extracellular DA are thought to be mediated by the activation of both D1-like and D2-like receptors. The former are believed to be critical for the DA component of methamphetamine-induced behavioural sensitization [97]. D1, dopamine receptor type-1; D2, dopamine receptor type-2; DAT, dopamine transporter; GERL, Golgi apparatus, endoplasmic reticulum and lysosomal system; MAO-A, monoamine oxidase type-A; VMAT-2, vesicular monoamine transporter type-2.

How METH acts is quite specific and involves three main targets. Briefly, METH produces (i) a disruption to physiological DA and NE vesicular storage, by interfering with the vesicular monoamine transporter type-2 (VMAT-2), (ii) it impairs the membrane DA transporter (DAT) [74] and NET [75], (iii) it competitively inhibits intracellular DA and NE metabolism by monoamine oxidase (MAO)-A [76] which are placed within both DA and NE terminals [77] (Fig. **2**).

METH acts on synaptic vesicles in several ways. It:

1. produces an altered proton gradient through the DA/NE storing vesicles, which makes DA readily diffusible from the vesicle to the cytoplasm [78].
2. directly inhibits the VMAT-2 [79, 80], which prevents DA and NE from entering the vesicles.
3. promotes DA and NE efflux from the vesicles to the cytoplasm [81];
4. moves and misplaces VMAT-2 from vesicles to non-physiological compartments, making the vesicles unable to store catecholamines [82, 83], leading to a “bizarre” release which is not related to physiological sites [84].

The effects of METH on catecholamine-storing vesicles produce massive amounts of free cytosolic DA and NE. This plays a key role in both the behavioural and neurotoxic effects of METH intake/administration. The increase in cytosolic DA and NE is accompanied by additional effects which transport massive levels of DA and NE from cytosol to the extracellular space. In fact, large amount of cytosolic catecholamines pass the plasma membrane either by passive diffusion or through a pathological reversion of the transporter [74, 85, 86]. Massive extracellular levels are enhanced by the concomitant inhibition of DA and NE metabolism and uptake (inhibition of MAO, and inhibition of DAT and NET, respectively). Inhibition of metabolism is due to the inhibitory effects of METH on MAO enzymes. Both MAO subtypes are involved, although the effects on MAO-A, which are present within DA and NE terminals, are predominant, while MAO-B are mainly placed in the glial cells [77, 87-92]. 

In DA terminals, DA is metabolized by MAO-A to the aldehyde 3,4-dihydroxyphenylacetaldehyde (DOPALD), which is rapidly converted by further oxidation to the acid 3,4-dihydroxyphenylacetic acid (DOPAC). On the other hand, in NE terminals the aldehyde produced by MAO-A from NE is preferentially reduced to the corresponding alcohol (which is in fact a glycol, 3,4-dihydroxyphenylethyleneglycol, DOPEG). This slight difference might impact on neurotoxicity since DOPALD is much more toxic than DOPEG. In spite of that, the effects of METH can be assumed the same when considering intracellular NE and DA since the selective and powerful inhibition of MAO-A is presumed to alter similarly the oxidative deamination of both DA and NE within pre-synaptic terminals. On the other hand, the Ki for NET is 10 fold lower than the Ki for DAT, which leads to 10 fold higher effects of METH on NE compared with DA uptake blockade [72]. 

Such a difference in the uptake mechanism in the presence of similar MAO inhibition is expected to produce higher extracellular NE compared with DA levels, as demonstrated by seminal papers [72]. This is critical in brain areas such as the PFC in which the numbers of DA and NE terminals are comparable. In this case, NE may have a leading role in sustaining the behavioural effects produced by METH administration [72]. This also occurs partly in the ventral striatum and specific limbic regions, which are rich in both DA and NE axon terminals [67, 93-96].

Turning now to the dorsal striatum, the critical effects of METH are almost exclusively produced on DA terminals and thereby on DA levels. In fact, striatal DA terminals are far in excess compared with NE terminals (roughly 100:1, [25]). The increase in striatal extracellular DA levels goes along with greater diffusion properties to extra-synaptic sites since the uptake (which limits diffusion) is strongly inhibited. Thus, METH produces a striatal scenario featuring a high amount of extracellular DA which spread over a greater distance for a prolonged amount of time. This leads to high and persistent “peaks” in DA levels, which are reminiscent of what occurs during L-DOPA administration in advanced stages of Parkinson’s disease [97].

## 5 METHAMPHETAMINE-INDUCED BEHAVIOURAL EFFECTS

Methamphetamine is known to profoundly affect behavior in humans and in a variety of animal species [98]. Behavioural effects on animals reflect those seen in humans, and are dose-dependent. Low doses of amphetamines increase locomotor activity, such as exploratory behaviour with rearing and sniffing.

Higher or repeated doses switch motor activity from locomotion to repetitive “in place” movements such as automatisms and stereotypies (licking, biting, gnawing). The higher the dose of amphetamines, the earlier the behavioural switch from hyperlocomotion to stereotypies. Moreover, the time spent in stereotypies increases with the dose of amphetamines (these points were extensively reviewed by Seiden* et al., *[98]). There is thus a different pattern of behavioural effects when METH is administered repeatedly. These behavioural changes mainly consist of exaggerated motor response to moderate doses of METH and above all depend on changes in the receptor signalling at a post-synaptic level.

These effects are produced both directly by NE and *via *modulating the massive DA release. The role of NE emerged in the last decade adding to well-established evidence showing that METH-induced behavioural changes are predominantly due to a robust DA release. In fact, selective damage to DA terminals in the dorsal striatum by 6-hydroxydopamine (6-OHDA) decreases stereotypies and increases locomotor activity induced by amphetamines [99-101], whereas selective damage to DA terminals within the nucleus accumbens (NAc) decreases hyper-locomotion [102]. This suggests that DA in NAc mediates hyperlocomotion, whereas DA in the dorsal striatum mediates stereotypies. In fact selective injections of amphetamine within the NAc [103] as well as in the ventrolateral striatum [104] induce hyper-locomotion, whereas injections in the dorsal striatum produce stereotypies [104-106].

Robust DA release is thus important to produce those behavioural effects occurring immediately after METH intake (acute behavioural effects) and long-term behavioural changes which reflect persistent alterations in some brain areas after repeated exposure to METH (behavioural sensitization). 

## 6 THE ROLE OF NE IN METHAMPHETAMINE-INDUCED BEHAVIOURAL CHANGES

The possibility that NE might be involved in the behavioural effects of psychostimulants was first hypothesized in the early 1960s [107, 108]. This was then supported by studies which found that LC and the dorsal NE bundle are targets of self-stimulation [109-111]. This self-stimulation takes place with a higher threshold in the presence of amphetamines [108, 112, 113]. These results led to the idea that NE mediates some effects of psychostimulants on intracerebral stimulation. 

A number of subsequent studies did not confirm this role of NE [113-115]. Thus, starting in the 1970s, DA, rather than NE, was generally accepted as the brain’s primary “reward neurotransmitter”. However, later studies demonstrated a dissociation between DA release and behavioural responses to amphetamines. 

We will thus now look at the effects played by of LC NE on METH-induced behavioural changes. The effects of METH interacting with NE can be divided into direct effects produced by NE and indirect effects due to the influence of NE on DA system

### 6.1 The Role of NE *per se*

In this context, it needs to be remarked that NE is massively released following METH administration, being METH-induced NE release in selected brain areas higher than DA release [72]. This makes it likely that NE plays a direct and important role in METH-induced behavioral changes. 

In particular, the amount of an oral dose of psychostimulants, which produce amphetamine-type subjective effects in humans, correlates with the potency of these psychostimulants in releasing NE, but not DA, thus suggesting that in humans NE plays a key role in METH addiction [72]. Accordingly, when evaluating the behavioural effects induced by a selective NET blocker in animals, this may reproduce the effects of amphetamines [116]. Lending further implications to these findings, some works have emphasized the role of increased NE release in producing sensitization following METH administration [73].

### 6.2 NE/DA Interactions 

NE activity is critical in regulating the effects of METH on DA neurons encompassing biochemistry, behaviour and neurotoxicity. As reviewed in Section 3, there are a number of brain sites in which NE-DA interactions take place. Areas critical for psychostimulant-induced behaviours, including the NAc, VTA, and PFC, receive a robust LC-NE innervation which alters DA release both in baseline, and above all, following METH administration. 

Classic studies on the role of NE on the activity of nigrostriatal DA neurons (reviewed in [117]) demonstrate that endogenous NE affects DA cells at the nigral level by modulating DA neuron firing and DA release after stimulation. In line with this, NE-containing varicosities arising from the LC project through the SN pars compacta [33, 118], and there can be a significant loss of nigral NE after LC damage in mice [26].

The effect of NE on the DA system involves ARs onto DA neurons. In particular, alpha1 ARs are may be critical in certain contingencies involved in the trans-synaptic effects which control the activity of DA neurons in response to D-amphetamine [119].

In fact, the alpha1 AR antagonist prazosin reduces amphetamine-induced hyperlocomotion and sensitization [120-128], possibly through the involvement of the alpha1b AR subtype [124, 127, 128]. The reduced behavioural response to METH found in alpha1b AR knock out (KO) mice is accompanied by a reduced DA release [122, 123, 129] and absence of DA neurotoxicity [130]. Conversely, transgenic mice overexpressing either wild-type or constitutively active alpha1b ARs show nigrostriatal damage associated with serious locomotor dysfunction, tremor at rest, epileptic seizures, and autonomic dysfunctions [131].

The response to psychostimulants produced by modulating the alpha1 AR-mediated NE activity contrasts with data showing an enhanced response to amphetamines found in NE-lacking animals [125, 132, 133].

These findings indicate that specific subtypes of ARs may have contrasting effects on DA neurons. It is thus critical to establish what net influence is produced by NE on the basis that changes in the expression or efficacy of receptor subtypes may lead to unexpected results. The role of ARs in modulating the DA-induced behaviour needs to be clarified and studies are underway to understand the fine mechanisms that regulate the AR-mediated response to psychostimulants.

Regarding mesencephalic DA neurons, NE also modulates VTA DA neurons [53, 54, 119] and there is evidence that NE-containing axons directly project to the VTA and NAc. Thus, it is likely that NE is important for ventral striatal DA release as well. In fact, micro-infusions of either DA or NE directly into the NAc similarly stimulate locomotor activity in rats [134, 135]. In addition to ventral striatum, NE terminals in the PFC play a key role in modulating amphetamine-induced DA release [136], which is critical in rewarding effects, and in reinforcing the behaviour and patterns of compulsive intake by amphetamines [137].

### 6.3 The Net Effects of NE System on Mesencephalic DA Neurons

**Fig. (3):**
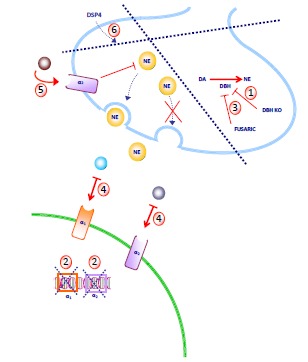
**Experimental tools to suppress/reduce NE activity in the brain.** Several experimental approaches can be exploited to suppress/reduce NE activity: 1) genetic ablation of dopamine beta-hydroxylase (DBH), the gene responsible for the conversion of DA into NE; 2) genetic ablation of the genes encoding for NE receptors (alpha1 or alpha2); 3) pharmacological inhibition of DBH by fusaric acid; 4) pharmacological blockade of alpha1 or alpha2 NE receptors; 5) inhibition of NE release by stimulation of pre-synaptic alpha2 receptors; 6) degeneration of locus coeruleus neurons induced by DSP-4. Only the latter mechanism causes the anatomical degeneration of NE neurons specifically arising from locus coeruleus, whereas the other approaches produce a suppression/reduction of NE activity in all brain areas receiving inputs from all NE nuclei. a1, alpha1-adrenoceptor; a2, alpha2-adrenoceptor; DA, dopamine; DBH, dopamine beta-hydroxylase; DBH KO, DBH knock out; NE, norepinephrine.

Experimental studies aimed at elucidating the role of NE on METH-induced behavioural effects are partly based on animal models involving specific damage to NE systems (Fig. **3**). This systemical approach by-passes the specific effects played by each receptor subtype, and indicates the net effect. 

**Fig. (4):**
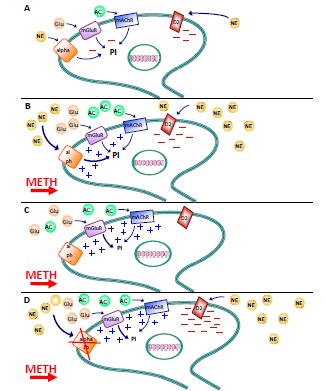
**Effects of NE input on activity of ventral midbrain DA neurons.** The figure shows a mesencephalic DA neuron. The main receptors modulating its activity are reported. The final effect on the DA neuron is the result of the degree/type of activation of each receptor subtype depending on the levels of their endogenous ligands in the extracellular space. **A**) **Basic conditions:** NE produces a long-lasting, tonic inhibition *via* activation of D2 DA autoreceptors placed on neuronal dendrites. At the same time, a short-lasting inhibition of these neurons is produced by metabotropic glutamate receptors (mGluRs), muscarinic receptors (mAChRs), or alpha1 adrenoceptors. These latter three receptor subtypes produce phosphoinositide (PI) hydrolysis. **B**) **METH administration with intact NE fibers:** METH causes a robust NE release from axon terminals, and D2 and alpha1 adrenoceptors are activated by the excess of extracellular NE. Prolonged activation of alpha1 adrenoceptors depolarizes DA cells directly and indirectly via cross-desensitization of the inhibitory mGluRs and mAChRs. This occurs due to the convergence of these receptors to produce the hydrolysis of PI, which in the presence of METH is facilitated by the simultaneous release of NE, glutamate and acetylcholine. However, NE significantly reduces the stimulation of DA neurons by the concomitant activation of D2, which inhibits the firing activity of DA neurons. Therefore, the final effect on DA neurons of such NE release is inhibitory. The prevalence of D2-mediated inhibition over alpha1 adrenoceptors activation is supported by data showing that in the absence of NE, increased methamphetamine-induced DA release occurs [65, 132]. **C**) **METH administration after NE depletion/lesion:** When METH administration occurs in the absence of NE (induced either by LC fiber lesion or NE synthesis inhibition) the final effect on DA neurons is an enhanced activation, since these neurons are no longer restrained by D2 stimulation by endogenous NE. **D**) **METH administration in the absence of alpha1 adrenoceptors activity:** Alpha1 adrenoceptors antagonists (e.g. prazosin) or knocking out alpha1b adrenoceptors suppress METH-induced DA neuron activation, as testified by a reduction in DA release [129]. Along with this, METH-induced behavioural effects and DA neurotoxicity are prevented [130]. This is further confirmed by the finding that mice overexpressing alpha1b adrenoceptors are characterized by spontaneous nigrostriatal DA toxicity [131]. Alpha1, alpha1 adrenoceptor; Alpha1b, alpha1b adrenoceptors; D2, D2 dopamine receptor; mAChR, muscarinic receptor; METH, methamphetamine; mGluR, metabotropic glutamate receptor; PI, phosphoinositide pathway.

Norepinephrine tonically inhibits the firing of DA neurons [138]. This occurs through the activation of D2 DA receptors, which work as inhibitory autoreceptors on midbrain DA neurons (Fig. **4A**) [139]. It is thus not surprising that in the absence of NE, the firing rate of these neurons is enhanced and the effects of METH on the mesolimbic (and mesostriatal) system are potentiated (Fig. **4**). Thus the prevalent role of D2 DA receptors explains why in baseline conditions the net effects of NE consists in inhibiting DA neurons (Fig. **4A** and **4B**) [119]. This explains why increased sensitivity of mesencephalic DA neurons to METH following damage to LC occurs.

In fact administration of METH in LC-damaged mice, enhances pulsatile DA release and motor stereotypies [65]. This is confirmed by the enhanced response to METH which occur in mice carrying a reversible (fusaric acid) and irreversible (DA beta-hydroxylase, DBH, KO mice) NE deficiency (Fig. **3**). This may lead to dramatic behavioural effects such as typical limbic seizures ([132], and our unpublished data). It is likely that METH-induced glutamate release in limbic brain regions may reach the threshold to trigger convulsive seizures in the absence of endogenous NE. In fact, METH is a powerful glutamate releaser [67-69], while NE is considered to be a pivotal endogenous seizure suppressive mechanism [20, 140-143]. 

## 7 METHAMPHETAMINE-INDUCED DOPAMINERGIC TOXICITY

The earliest neurochemical effect of amphetamine derivatives is a robust release of the monoamines DA, NE, and serotonin [66]. This leads to a fast depletion of these neurotransmitters, which are drastically reduced in the extracellular compartment just a few hours after the administration of amphetamines [65, 132]. When a single high dose or repetitive low doses of amphetamines derivatives are administered, early acute effects are followed by long-term neurotoxic effects, consisting of damage to DA axonal terminals [144-149].

In both mice and rats, acute repeated injections of METH produce long-lasting decreases in DA levels [145, 149-153], and long-term reductions in several DA markers, such as TH [144, 154, 155], DAT [152, 156, 157], and VMAT-2 [155, 157-159] within the striatum. Morphological studies demonstrate that the persistent loss of integrity of DA biochemical markers is due to the degeneration of DA axon terminals [145-149].

Several studies suggest that the mobilization of endogenous DA plays a key role in producing METH-induced damage to DA axons. In fact, inhibition of DA synthesis prevents METH-induced DA damage [144, 160-162], while treatments that increase cytoplasmic DA levels exacerbate METH neurotoxicity [160, 163, 164].

METH toxicity is thus related to the molecular effects of amphetamines on DA terminals due to effects on DA vesicles, MAO-A and DAT (Fig. **2**). These produce massive amounts of axoplasmic DA, followed by a massive efflux in the extracellular space. The causal link between cytosolic DA levels and METH toxicity is supported by several studies. In fact, overexpression of VMAT2, which stores DA in the vesicles, significantly protects PC12 against METH toxicity [165], while a mutant strain of mice, which possesses only 5-10% of the VMAT2 expressed by wild-type animals, undergo enhanced METH-induced striatal neurotoxicity [166]. For the same reasons, DAT inhibitors protect against METH-induced striatal DA damage [74, 83, 167]. Similar protective effects have been described in the striatum of DAT KO mice [168]. Finally, competitive inhibition of MAO-A increases METH-induced DA [163], while MAO KOs are protected against DA toxicity [77].

When free DA is elevated into the cytoplasm in the presence of MAO inhibition, auto-oxidation leads to the formation of hydrogen peroxide, superoxide radicals and DA quinones [78, 169-172]. These highly reactive compounds trigger a cascade of oxidative reactions leading to irreversible damage in lipids, proteins and organelles [173, 174] within the DA axon terminals and surrounding compartments. In fact, we measured an increase in reactive oxygen species that was associated with the progressive augmentation of DA in METH-treated mice [175]. A variety of mechanisms and molecules are implicated in METH-induced DA axon damage, although the role of DA remains well established.

## 8 THE ROLE OF NE ON METHAMPHETAMINE-INDUCED DOPAMINERGIC TOXICITY

Enhancement of METH-induced behavioural effects in the absence of NE extends to neurotoxicity. Experimental damage to LC neurons exacerbates DA degeneration induced by amphetamines by (1) enhancing the DA neurotoxic damage, and (2) by reducing the threshold for inducing DA neurotoxic damage. The hypothesis that LC NE neurons protect DA neurons was demonstrated following administration of different amphetamine derivatives [65, 132, 176-178]. In addition, both in mice and rats, LC lesion makes toxic a small dose of METH, not sufficient by itself to produce a nigrostriatal lesion [25, 26].

In keeping with this, animals carrying an inborn NE hyperinnervation of target areas are resistant to DA neurotoxicity [179]. Similarly, pharmacological stimulation of NE neurons provides protection from METH-induced DA neurotoxicity [65].

In order to shed light on whether this increased vulnerability of DA neurons in the presence of a lesion of LC is achieved *via *an acute enhancement of METH toxicity and/or through an impairment of the recovery of the nigrostriatal DA pathway, several different methodological approaches have been used in different animal models. Using intrastriatal microdialysis in rodents, it was found that NE loss enhances the amount of DA released by the nigrostriatal axon terminals after METH [65], demonstrating that LC lesion makes nigrostriatal DA neurons more sensitive to the early acute neurotoxic events induced by METH.

This effect does not depend on the prolonged persistence of METH in the striatum of LC-lesioned animals. In NE-damaged mice, the striatal concentration peak observed one hour after METH injection was unmodified [177]. This appears to rule out NE terminals acting as buffers, which in turn could decrease the availability of METH to DA neurons [64, 180, 181].

As originally postulated in [182], the influence of the LC on the nigrostriatal DA pathway may also involve a cortico-striatal loop. Cortico-striatal fibers are the main glutamatergic input to the neostriatum, and increased striatal glutamate sustains a deleterious effect on nigrostriatal DA axon terminals [68, 183].

Effects extending beyond NE-DA interaction should also be considered. In fact, NE affects the microglial release of specific cytokines [23, 24] and it has been shown that these compounds modulate the viability of mesencephalic neurons [184].

Interestingly, enhancement of METH neurotoxicity is achieved by loss of NE innervation and not only by loss of NE itself. We addressed this aspect by comparing the effects of METH in mice with NE lesions achieved by DSP-4 administration and those with intact NE terminals but specifically lacking NE due to genetic KO or acute pharmacological blockade of the NE biosynthetic enzyme DBH [132]. Dopamine beta-hydroxylase KO and fusaric-acid-treated mice have anatomically intact NE innervation, though are NE deficient. This fact should not be underestimated, since NE terminals contain several co-transmitters, such as adenosine, neuropeptide Y, and galanin, which may modulate METH-induced toxicity. NE co-transmitters, such as galanin, inhibit DA release [185-187].

It is likely that both NE and its co-transmitters are critical since the absence of NE alone, as occurs in DBH KO mice or intact mice, which when treated with fusaric acid, require multiple METH administration to magnify the effects of METH. On the other hand, the anatomical loss of integrity of NE terminals, which is expected to produce a deficiency of both NE and its co-transmitters, worsened the effects of METH already after the first administration [132]. 

Morphological analysis of striatum in these models provides intriguing results. When observed by electron microscopy, we found membranous multilayer whorls within striatal neurons of NE-depleted mice also in the absence of METH, thus indicating that loss of NE innervation does affect the ultrastructural morphology of striatal neurons [132]. 

## CONCLUSIONS

We have reviewed the role of the NE neurons of LC in mediating the behavioural and neurotoxic effects induced by methamphetamine in the light of recent literature that has reported novel anatomical, electrophysiological, biochemical, behavioural and toxicity data. A variety of morphological connections explain the powerful effects of LC NE neurons on brain areas mediating METH-induced behavioural alterations. These connections justify increasing evidence that highlights that NE is an important neurotransmitter involved in methamphetamine-induced motor alterations and addiction. This concept is amplified by the occurrence of multiple reciprocal connections between NE and DA neurons both at the level of the perikaria (i.e. within mesencephalon and pons) and within target regions, such as the ventral and dorsal striatum, PFC, and several limbic allocortical and nuclear sites. In fact, apart from highlighting that NE plays a direct role in mediating METH-induced behavioural alterations, we believe that this review demonstrates that LC-NE neurons are powerful modulators of the DA system, which plays a pivotal role in mediating the effects of METH in the CNS. This concept is extended to METH-induced neurotoxicity, which is mainly produced against DA-containing neurons and is triggered by DA itself. The modulatory effects of NE on DA availability eventually lead to a powerful effect in modulating METH-induced neurotoxicity. The physiological mechanisms involved and receptor subtypes recruited by these interactions were considered in discussing this topical issue.

## Figures and Tables

**Fig. (1):**
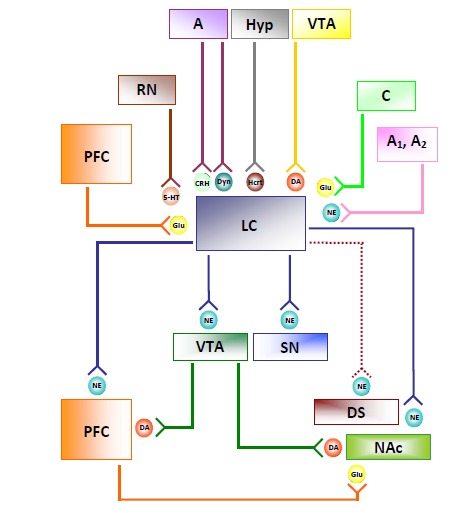
**Locus coeruleus projections to brain areas responsible for amphetamines-induced behavioural responses.** Amphetamines cause a massive NE release in all the brain areas that receive NE inputs from locus coeruleus. Locus coeruleus inputs into NAc are suggested by much experimental evidence (see text). An increase in NE-mediated signals on NAc and PFC produces the typical behavioural responses induced by amphetamine derivatives. Conversely, dorsal striatum possesses only scattered NE afferents from locus coeruleus. Locus Coeruleus receives inputs from several cortical and subcortical regions. Descending fibers from cortical areas mainly derive from the dorsolateral and dorsomedial prefrontal cortex. Amigdalo-coeruleus projections releasing corticotropin-releasing hormone and dynorphin suggest the involvement of LC in the control of limbic functions. Hypocretin/orexin innervation from the posterior hypothalamus is involved in the LC regulation of sleep/arousal responses. Finally, monoaminergic innervation to LC derives from raphe nuclei, lower medullary A1 and A2 NE nuclei, and VTA. A, amigdala; A1, A2, NE nuclei; C, cerebellum; CRT, corticotropin-releasing hormone; DA, dopamine; DS, dorsal striatum; Glu, glutamate; Hcrt, hypocretin; Hyp, hypothalamus; 5-HT, serotonin; NAc, nucleus accumbens; NE, norepinephrine; LC, locus coeruleus; PFC, prefrontal cortex; RN, raphe nuclei; SN, substantia nigra; VTA, ventral tegmental area.

## References

[1] Dahlstrom A,  Fuxe K (1964). Evidence for the existence of monoamine
neurons in the central nervous system. I. Demonstration of
monoamines in the cell bodies of brain stem neurons. Acta Physiol. Scand.

[2] Russell GV (1955). The nucleus locus coeruleus (dorsolateralis tegmenti). Tex. Rep. Biol. Med.

[3] Brodal A, Brodal A (1981). The reticular formation and some related nuclei. The
nucleus locus coeruleus. Neurological anatomy in relation to
clinical medicine.

[4] Aston-Jones G,  Shipley MT, Paxinos G (1995). The locus coeruleus, A5 and A7 noradrenergic cell groups. The rat nervous system.

[5] Halliday G, Paxinos G, Mai JK (2004). Substantia nigra and locus Coeruleus. The Human
Nervous System - II Edition.

[6] Aston-Jones GS, Iba M,  Clayton E,  Rajkowski J,  Cohen J, Ordway GA, Schwartz MA, Frazer A (2007). Locus coeruleus and regulation of behavioral flexibility and
attention: clinical implications. Brain Norepinephrine -
Neurobiology and Therapeutics.

[7] Ward DG,  Gunn CG (1976). Locus coeruleus complex: elicitation of a pressor response and a brainstem region necessary for its occurrence. Brain Res.

[8] Olszewski J,  Baxter D (1954). Cytoarchitecture of the human brain
stem.

[9] Iversen LL,  Rossor MN,  Reynolds GP,  Hills R,  Roth M,  Mountjoy CQ,  Foote SL,  Morrison JH,  Bloom FE (1983). Loss of pigmented dopamine-beta-hydroxylase positive cells from locus coeruleus in senile dementia of Alzheimer’s type. Neurosci. Lett.

[10] Baker KG,  Toerk I,  Hornung JP,  Halasz P (1989). The human locus coeruleus complex: an immunohistochemical and three-dimensional reconstruction study. Exp. Brain. Res.

[11] Vijayashankar N,  Brody HA (1979). Quantitative study of the pigmented neurons in the locus coeruleus and sub-coeruleus in 
man as related to ageing. J. Neuropathol. Exp. Neurol.

[12] German DC,  Walker BS,  Manaye K,  Smith WK,  Woodward DJ,  North AJ (1988). The human locus coeruleus: computer reconstruction of cellular distribution. J. Neurosci.

[13] Nagai T,  Satoh K,  Immamoto K,  Maeda T (1981). Divergent projections of catecholamine neurons of the locus coeruleus as revealed by fluorescent retrograde double labeling technique. Neurosci. Lett.

[14] Room P,  Postema F,  Korf J (1981). Divergent axon collaterals of rat locus coeruleus neurons: demonstration by a fluorescent double-labeling technique. Brain Res.

[15] Fornai F, Ordway GA, Schwartz MA, Frazer A (2007). Norepinephrine in neurological disorders. Brain
Norepinephrine - Neurobiology and Therapeutics.

[16] Simpson KL,  Lin RCS, Ordway GA, Schwartz MA, Frazer A (2007). Neuroanatomical and chemical
organization of the locus Coeruleus. Brain Norepinephrine -
Neurobiology and Therapeutics.

[17] Moises HC,  Waterhouse BD,  Woodward DJ (1981). Locus coeruleus stimulation potentiates Purkinje cell responses to afferent input: the climbing fiber system. Brain Res.

[18] Nieuwenhuys JV,  Voogd J,  vanHuijzen C (1988). The human central
nervous system: a synopsis and atlas.

[19] Fornai F,  di Poggio AB,  Pellegrini A,  Ruggieri S,  Paparelli A (2007). Norepinephrine in Parkinson’s disease: from disease progression to current therapeutics. Curr. Med. Chem.

[20] Fornai F,  Ruffoli R,  Giorgi FS,  Paparelli A (2011). The role of locus coeruleus in the antiepileptic activity induced by vagus nerve stimulation. Eur. J. Neurosci.

[21] Harik SI,  McGunigal Jr T (1984). The protective influence of the 
locus coeruleus on the blood-brain barrier. Ann. Neurol.

[22] Stone EA,  Ariano MA (1989). Are glial cells targets of the central noradrenergic system? A review of the evidence. Brain Res. Rev.

[23] Norris JG,  Benveniste EN (1993). Interleukin-6 production by astrocytes: induction by the neurotransmitter norepinephrine. J. Neuroimmunol.

[24] Kingham PJ,  Pocock JM (2000). Microglial apoptosis induced by chromograinin A is mediated by mitochondrial depolarisation and the permeability transition but not by cytochrom C release. J. Neurochem.

[25] Fornai F,  Bassi L,  Torracca MT,  Alessandrì MG,  Scalori V,  Corsini GU (1996). Region- and neurotransmitter-dependent species and strain differences in DSP-4-induced monoamine depletion in rodents. Neurodegeneration.

[26] Fornai F,  Torracca MT,  Bassi L,  D’Errigo DA,  Scalori V,  Corsini GU (1996). Norepinephrine loss selectively enhances chronic nigrostriatal dopamine depletion in mice and rats. Brain Res.

[27] Soldani P,  Fornai F (1999). The functional anatomy of noradrenergic neurons in Parkinson’s disease. Funct. Neurol.

[28] Falck H,  Hillarp NA,  Thieme G,  Torp A (1962). Fluorescence of catecholamines and related compounds condensed with formaldehyde. J. Histochem. Cytochem.

[29] Levitt P,  Moore RY (1979). Origin and organization of brainstem catecholamine innervation in the rat. J. Comp. Neurol.

[30] Fuxe K,  Hoekfelt T,  Ungerstedt U, Pfeifer CC, Smythies JR (1970). Morphological and functional aspects of central monoamine neurons. Int. Rev.
Neurobiol. 13.

[31] Maeda T,  Shimizu N (1972). Projections ascendantes du locus coeruleus et d'autres neurones aminergiques pontiques au niveau du prosencephale du rat. Brain Res.

[32] Rinvik E,  Grofova I,  Hammond C,  Deniau JM,  Feger J, Poirier LJ, Sourkes TL, Bedard PJ (1979). Afferent connections to the subthalamic nucleus in the monkey and
the cat studied with the HRP technique. The extrapyramidal
system and its disorders.

[33] Collingridge GL,  James TA,  MacLeod NK (1979). Neurochemical and electrophysiological evidence for a projection from the locus coeruleus to the substantia nigra. J. Physiol.

[34] Jones BE,  Moore RY (1977). Ascending projections of the locus
coeruleus in the rat. II Autoradiographic study. Brain Res.

[35] Olson L,  Fuxe K (1971). On the projections from the locus coeruleus norepinephrine neurons: the cerebellar innervation. Brain Res.

[36] Olson L,  Fuxe K (1972). Further mapping out of central norepinephrine neuron systems: projections of the subcoeruleus area. Brain Res.

[37] Fulceri F,  Biagioni F,  Lenzi P,  Falleni A,  Gesi M,  Ruggieri S,  Fornai F (2006). Nigrostriatal damage with 6-OHDA: validation 
of routinely applied procedures. Ann. N.Y. Acad. Sci.

[38] Mizuno N,  Nakamura Y (1970). Direct hypothalamic projections to the locus coeruleus. Brain Res.

[39] Snider RS (1975). A cerebellar-ceruleus pathway. Brain Res.

[40] Sakai K,  Touret M, Salvert D,  Leger L,  Jouvet M (1977). Afferent projections to the cat locus coeruleus as visualized by the horseradish peroxidase tecnique. Brain Res.

[41] Hopkins DA,  Holstege G (1978). Amygdaloid projections to the mesencephalon, pons, medulla oblongata in the cat. Exp. Brain Res.

[42] Cedarbaum JM,  Aghajanian GK (1978). Afferent projections to the rat locus coeruleus as determined by a retrograde tracing technique. J. Comp. Neurol.

[43] Beckstead RM,  Domesick VB,  Nauta WJH (1979). Efferent connections of the substantia nigra and ventral tegmental area in the rat. Brain Res.

[44] Arnsten A,  Goldman-Rakic PS (1984). Selective prefrontal cortical projections to the region of the locus coeruleus and raphe nuclei in the rhesus monkey. Brain Res.

[45] Deutch AY,  Goldstein M,  Roth RH (1986). Activation of the locus coeruleus induced by selective stimulation of the ventral tegmental area. Brain Res.

[46] Berridge CW,  Stratford TL,  Foote SL,  Kelley AE (1997). Distribution of dopamine beta-hydroxylase-like immunoreactive fibers within the shell subregion of the nucleus accumbens. Synapse.

[47] Schroeter S,  Apparsundaram S,  Wiley RG,  Miner LH,  Sesack SR,  Blakely RD (2000). Immunolocalization of the cocaine- and antidepressant-sensitive l-norepinephrine transporter. J. Comp. Neurol.

[48] Delfs JM,  Zhu Y,  Druhan JP,  Aston-Jones GS (1998). Origin of noradrenergic afferents to the shell subregion of the nucleus accumbens: anterograde and retrograde tract-tracing studies in the rat. Brain Res.

[49] Park J,  Aragona BJ,  Kile BM,  Carelli RM,  Wightman RM (2010). *In vivo* voltammetric monitoring of catecholamine release in subterritories of the nucleus accumbens shell. Neuroscience.

[50] Jones BE,  Halaris AE,  McIlhany M,  Moore RY (1977). Ascending
projections of the locus coeruleus in the rat. I. Axonal transport in
central norepinephrine neurons. Brain Res.

[51] Fallon JH,  Koziell DA,  Moore RY (1978). Catecholamine
innervation of the basal forebrain. II. Amygdala, suprarhinal cortex
and entorhinal cortex. J. Comp. Neurol.

[52] Simon H,  Le Moal M,  Stinus L,  Calas A (1979). Anatomical relationships between the ventral mesencephalic tegmentum-A 10 region and the locus coeruleus as demonstrated by anterograde 
and retrograde tracing techniques. J. Neural. Transm.

[53] Grenhoff J,  Nisell M,  Ferre S,  Aston-Jones G,  Svensson TH (1993). Noradrenergic modulation of midbrain dopamine cell firing elicited by stimulation of the locus coeruleus in the rat. J. Neural. Transm. Gen. Sect.

[54] Grenhoff J,  Svensson TH (1993). Prazosin modulates the firing pattern of dopamine neurons in rat ventral tegmental area. Eur. J. Pharmacol.

[55] Jones LS,  Gauger LL,  Davis JN (1985). Anatomy of brain alpha 1-adrenergic receptors: *in vitro* autoradiography with [125I]-heat. J. Comp. Neurol.

[56] Lee A,  Wissekerke AE,  Rosin DL,  Lynch KR (1998). Localization of alpha2Cadrenergic receptor immunoreactivity in catecholaminergic neurons in the rat central nervous system. Neuroscience.

[57] Mejías-Aponte CA,  Drouin C,  Aston-Jones G (2009). Adrenergic and Noradrenergic Innervation of the Midbrain Ventral Tegmental 
Area and Retrorubral Field: Prominent Inputs from Medullary Homeostatic Centers. J. Neurosci.

[58] Liprando LA,  Miner LH,  Blakely RD,  Lewis DA,  Sesack SR (2004). Ultrastructural interactions between terminals expressing the norepinephrine transporter and dopamine neurons in the rat and monkey ventral tegmental area. Synapse.

[59] Hertel P,  Nomikos GG,  Svensson TH (1999). Idazoxan preferentially increases dopamine output in the rat medial prefrontal cortex at the nerve terminal level. Eur. J. Pharmacol.

[60] Kitanaka N,  Kitanaka AJ,  Watabe K,  Takemura M (2010). Low-dose pretreatment with clorgyline decreases the levels of 3-methoxy-4-hydroxyphenylglycol in the striatum and nucleus accumbens and attenuates methamphetamine-induced conditioned place preference in rats. Neuroscience.

[61] Russell VA,  Lamm MC,  Allin R,  de Villiers AS,  Searson A,  Taljaard JJ (1989). Effect of selective noradrenergic denervation on norepinephrine content and [3H]dopamine release in rat nucleus accumbens slices. Neurochem. Res.

[62] Lategan AJ,  Marien MR,  Colpaert FC (1990). Effects of locus coeruleus lesions on the release of endogenous dopamine in the rat nucleus accumbens and caudate nucleus as determined by intracerebral microdialysis. Brain Res.

[63] Lategan AJ,  Marien MR,  Colpaert FC (1992). Suppression of nigrostriatal and mesolimbic dopamine release *in vivo* following norepinephrine depletion by DSP-4: a microdialysis study. Life Sci.

[64] Fornai F,  Bassi L,  Bonaccorsi I,  Giorgi F,  Corsini GU (1997). Norepinephrine loss selectivity exacerbates nigrostriatal toxicity in different species of rodents. Funct. Neurol.

[65] Fornai F,  Alessandrì MG,  Torracca MT,  Bassi L,  Scalori V,  Corsini GU (1998). Noradrenergic modulation of methamphetamine-induced striatal dopamine depletion. Ann. N.Y. Acad. Sci.

[66] Berger UV,  Gu XF,  Azmitia EC (1992). The substituted amphetamines 3,4-methylenedioxymethamphetamine, methamphetamine, p-chloroamphetamine and fenfluramine induce 5-hydroxytryptamine release *via* a common mechanism blocked by fluoxetine and cocaine. Eur. J. Pharmacol.

[67] Abekawa T,  Ohmori T,  Koyama T (1994). Effects of repeated administration of a high dose of methamphetamine on dopamine and glutamate release in rat striatum and nucleus accumbens. Brain Res.

[68] Sonsalla PK,  Nicklas WJ,  Heikkila RE (1989). Role for excitatory amino acids in methamphetamine-induced nigrostriatal dopaminergic toxicity. Science.

[69] Nash JF,  Yamamoto BK (1992). Methamphetamine neurotoxicity 
and striatal glutamate release: comparison to 3,4-methylenedioxymethamphetamine. Brain Res.

[70] Tsai TH,  Chen CF (1994). Simultaneous measurement of acetylcholine and monoamines by two serial on-line microdialysis systems: effects of methamphetamine on neurotransmitters release from 
the striatum of freely moving rats. Neurosci. Lett.

[71] Northrop NA,  Smith LP,  Yamamoto BK,  Eyerman DJ (2011). Regulation of glutamate release by α7 nicotinic receptors: differential role in methamphetamine-induced damage to dopaminergic and serotonergic terminals. J. Pharmacol. Exp. Ther.

[72] Rothman RB,  Baumann MH,  Dersch CM,  Romero  
DV,  Rice KC,  Carroll FI,  Partilla JS (2001). Amphetamine-type central nervous system stimulants release norepinephrine more potently than they release dopamine and serotonin. Synapse.

[73] Yui K,  Goto K,  Ikemoto S (2004). The role of noradrenergic and dopaminergic hyperactivity in the development of spontaneous recurrence of methamphetamine psychosis and susceptibility to episode recurrence. Ann. N.Y. Acad. Sci.

[74] Schmidt CJ,  Gibb JW (1985). Role of the dopamine uptake carrier in the neurochemical response to methamphetamine: effects of amfonelic acid. Eur. J. Pharmacol.

[75] Han DD,  Gu HH (2006). Comparison of the monoamine transporters from human and mouse in their sensitivities to psychostimulant drugs. BMC Pharmacol.

[76] Suzuki O,  Hattori H,  Asano M,  Oya M,  Katsumata Y (1980). Inhibition of monoamine oxidase by d-methamphetamine. Biochem. Pharmacol.

[77] Fornai F,  Giorgi FS,  Gesi M,  Chen K,  Alessandrì MG,  Shih JC (2001). Biochemical effects of monoamine neurotoxins DSP-4 and MDMA in specific brain regions of MAO-B-deficient mice. Synapse.

[78] Cubells JF,  Rayport S,  Rajendran G,  Sulzer D (1994). Methamphetamine neurotoxicity involves vacuolation of endocytic organelles and dopamine-dependent intracellular oxidative stress. J. Neurosci.

[79] Ary TE,  Komiskey HL (1980). Phencyclidine: effect on the accumulation of 3H-dopamine in synaptic vesicles. Life Sci.

[80] Brown JM,  Hanson GR,  Fleckenstein AE (2000). Methamphetamine rapidly decreases vesicular dopamine uptake. J. Neurochem.

[81] Volz  J, Hanson GR,  Fleckenstein AE (2006). Kinetic analysis of developmental changes in vesicular monoamine transporter-2 function. Synapse.

[82] Sandoval V,  Riddle EL,  Hanson GR,  Fleckenstein AE (2002). Methylphenidate redistributes vesicular monoamine transporter-2: role of dopamine receptors. J. Neurosci.

[83] Sandoval V,  Riddle EL,  Hanson GR,  Fleckenstein AE (2003). Methylphenidate alters vesicular monoamine transport and prevents methamphetamine-induced dopaminergic deficits. J. Pharmacol. Exp. Ther.

[84] Fleckenstein AE,  Volz TJ, Riddle EL,  Gibb JW,  Hanson GR (2007). New insights into the mechanism of action of amphetamines. Annu. Rev. Pharmacol. Toxicol.

[85] Sulzer D,  Rayport S (1990). Amphetamines and other psychostimulants reduce pH gradients in midbrain dopaminergic neurons and chromaffin granules: a mechanism of action. Neuron.

[86] Sulzer D,  Sonders MS,  Poulsen NW,  Galli A (2005). Mechanisms of neurotransmitter release by amphetamines: a review. Prog. Neurobiol.

[87] Agid Y,  Javoy F,  Youdim MB (1973). Monoamine oxidase and aldehyde dehydrogenase activity in the striatum of rats after 6-hydroxydopamine lesion of the nigrostriatal pathway. Br. J. Pharmacol.

[88] Youdim MB (1974). Heterogeneity of rat brain mitochondrial monoamine oxidase. Adv. Biochem. Psychopharmacol.

[89] Fornai F,  Chen K,  Giorgi FS,  Gesi M,  Alessandri MG,  Shih JC (1999). Striatal dopamine metabolism in monoamine oxidase B-deficient mice: a brain dialysis study. J. Neurochem.

[90] Gesi M,  Santinami A,  Ruffoli R,  Conti G,  Fornai F (2001). Novel aspects of dopamine oxidative metabolism (confounding outcomes take place of certainties). Pharmacol. Toxicol.

[91] Tipton KF,  Boyce S,  O’Sullivan J,  Davey GP,  Healy J (2004). Monoamine oxidases: certainties and uncertainties. Curr. Med. Chem.

[92] Youdim MB,  Edmondson D,  Tipton KF (2006). The therapeutic potential of monoamine oxidase inhibitors. Nat. Rev. Neurosci.

[93] Stephans SE,  Yamamoto BY (1995). Effect of repeated methamphetamine administrations on dopamine and glutamate efflux in rat prefrontal cortex. Brain Res.

[94] Nishijima K,  Kashiwa A,  Hashimoto A,  Iwama H,  Umino  
A,  Nishikawa T (1996). Differential effects of phencyclidine and methamphetamine on dopamine metabolism in rat frontal cortex and striatum as revealed by *in vivo* dialysis. Synapse.

[95] Piccini P,  Pavese N,  Brooks DJ (2003). Endogenous dopamine release after pharmacological challenges in Parkinson’s disease. Ann. Neurol.

[96] Uehara T,  Sumiyoshi T,  Itoh H,  Kurachi M (2004). Inhibition of dopamine synthesis with alpha-methyl-p-tyrosine abolishes the enhancement of methamphetamine-induced extracellular dopamine levels in the amygdala of rats with excitotoxic lesions of the entorhinal cortex. Neurosci. Lett.

[97] Fornai F,  Biagioni F,  Fulceri F,  Murri L,  Ruggieri S,  Paparelli A (2009). Intermittent Dopaminergic stimulation causes behavioral sensitization in the addicted brain and parkinsonism. Int. Rev. Neurobiol.

[98] Seiden LS,  Sabol KE,  Ricaurte GA (1993). Amphetamine: effects on catecholamine systems and behavior. Annu. Rev. Pharmacol. Toxicol.

[99] Kelly PH,  Saviour PW,  Iversen SD (1975). Amphetamine and apomorphine responses in the rat following 6-OHDA lesions of the nucleus accumbens septi and corpus striatum. Brain Res.

[100] Costall B,  Naylor RJ, Ellinwood EH, Kilbey MM (1977). Mesolimbic and extrapyramidal sites for
the mediation of stereotyped behavior patterns and hyperactivity by
amphetamine and apomorphine in the rat. Cocaine and Other
Stimulants.

[101] Joyce EM,  Iversen SD (1984). Dissociable effects of 6-OHDA-
induced lesions of neostriatum on anorexia, locomotor activity 
and stereotypy: the role of behavioural competition. Psychopharmacology.

[102] Creese I,  Iversen SD (1974). A role of forebrain dopamine systems in amphetamine induced stereotyped behaviour in the rat. Psychopharmacology.

[103] Pijnenburg AJ,  Honig WM,  Van Der Heyden JA,  Van Rossum JM (1976). Effects of chemical stimulation of the mesolimbic dopamine system upon locomotor activity. Eur. J. Pharmacol.

[104] Kelley AE,  Lang CG,  Gauthier AM (1988). Induction of oral stereotypy following amphetamine microinjection into a discrete subregion of the striatum. Psychopharmacology.

[105] Makanjuola ROA,  Dow RC,  Ashcroft GW (1980). Behavioural responses to stereotactically controlled injections of monoamine neurotransmitters into the accumbens and caudate-putamen nuclei. Psychopharmacology.

[106] Staton DM,  Solomon PR (1984). Microinjections of d-amphetamine into the nucleus accumbens anda caudate-putamen differentially affect stereotypy and locomotion in the rat. Phsysiol. Psychol.

[107] Poschel BP,  Ninteman FW (1963). Norepinephrine: a possible excitatory neurohormone of the reward system. Life Sci.

[108] Stein L (1964). Self-stimulation of the brain and the central stimulant action of amphetamine. Fed. Proc.

[109] Dresse A (1966). Importance of the noradrenergic mesencephalotelencephalic system as an anatomic substrate of autostimulation behavior. Life Sci.

[110] Crow TJ,  Spear PJ,  Arbuthnott GW (1972). Intracranial selfstimulation with electrodes in the region of the locus coeruleus. Brain Res.

[111] Ritter S,  Stein L (1973). Self-stimulation of noradrenergic cell group (A6) in locus coeruleus of rats. J. Comp. Physiol. Psychol.

[112] Crow TJ (1970). Enhancement of coaine of intra-cranial selfstimulation in the rat. Life Sci.

[113] Wise RA (1978). Catecholamine theories of reward: a critical review. Brain Res.

[114] Amaral DG,  Routtenberg A (1975). Locus coeruleus and intracranial self-stimulation: a cautionary note. Behav. Biol.

[115] Simon H,  Le Moal M,  Cardo B (1975). Self-stimulation in the dorsal pontine tegmentum in the rat. Behav. Biol.

[116] Kamien JB,  Woolverton WL (1989). A pharmacological analysis of the discriminative stimulus properties of d-amphetamine in rhesus monkeys. J. Pharmacol. Exp. Ther.

[117] Weinshenker D,  Schroeder JP (2007). There and back again: a tale of norepinephrine and drug addiction. Neuropsychopharmacology.

[118] Mason ST,  Fibiger HT (1979). Regional topography within noradrenergic locus coeruleus as revealed by retrograde transport of horseradish peroxidase. J. Comp. Neurol.

[119] Paladini CA,  Fiorillo CD,  Morikawa H,  Williams JT (2001). Amphetamine selectively blocks inhibitory glutamate transmission in dopamine neurons. Nat. Neurosci.

[120] Snoddy AM,  Tessel RE (1985). Prazosin: effect on psychomotorstimulant cues and locomotor activity in mice. Eur. J. Pharmacol.

[121] Dickinson SL,  Gadie B,  Tulloch IF (1988). Alpha 1- and alpha 2-adrenoreceptor antagonists differentially influence locomotor 
and stereotyped behaviour induced by d-amphetamine and apomorphine in the rat. Psychopharmacology (Berl.).

[122] Blanc G,  Trovero F,  Vezina P,  Herve D,  Godeheu AM,  Glowinski J,  Tassin JP (1994). Blockade of prefronto-cortical alpha 1-adrenergic receptors prevents locomotor hyperactivity induced by subcortical D-amphetamine injection. Eur. J. Neurosci.

[123] Darracq L,  Blanc G,  Glowinski J,  Tassin JP (1998). Importance of the norepinephrine–dopamine coupling in the locomotor activating effects of D-amphetamine. J. Neurosci.

[124] Drouin C,  Darracq L,  Trovero F,  Blanc G,  Glowinski J,  Cotecchia S,  Tassin JP (2002). Alpha1b-adrenergic receptors control locomotor and rewarding effects of psychostimulants and opiates. J. Neurosci.

[125] Weinshenker D,  Miller NS,  Blizinsky K,  Laughlin ML,  Palmiter RD (2002). Mice with chronic norepinephrine deficiency resemble amphetamine-sensitized animals. Proc. Natl. Acad. Sci. USA.

[126] Wellman P,  Ho D,  Cepeda-Benito A,  Bellinger L,  Nation J (2002). Cocaine-induced hypophagia and hyperlocomotion in rats are attenuated by prazosin. Eur. J. Pharmacol.

[127] Auclair A,  Drouin C,  Cotecchia S,  Glowinski J,  Tassin JP (2004). 5-HT2A and alpha1b-adrenergic receptors entirely mediate dopamine release, locomotor response and behavioural sensitization to opiates and psychostimulants. Eur. J. Neurosci.

[128] Salomon L,  Lanteri C,  Glowinski J,  Tassin JP (2006). Behavioral sensitization to amphetamine results from an uncoupling between noradrenergic and serotonergic neurons. Proc. Natl. Acad. Sci. USA.

[129] Auclair A,  Cotecchia S,  Glowinski J,  Tassin JP (2002). D-Amphetamine fails to increase extracellular dopamine levels in mice lacking alpha 1b-adrenergic receptors: relationship between functional and nonfunctional dopamine release. J. Neurosci.

[130] Battaglia G,  Fornai F,  Busceti CL,  Lembo G,  Nicoletti F, De Blasi A (2003). Alpha-1B adrenergic receptor knockout mice are protected against methamphetamine toxicity. J. Neurochem.

[131] Zuscik MJ,  Sands S,  Ross SA,  Waugh DJ,  Gaivin RJ,  Morilak D,  Perez DM (2000). Overexpression of the alpha-1B-adrenergic receptor causes apoptotic neurodegeneration: multiple system atrophy. Nat. Med.

[132] Weinshenker D,  Ferrucci M,  Busceti CL,  Biagioni F,  Lazzeri G,  Liles LC,  Lenzi P,  Pasquali L,  Murri L,  Paparelli A,  Fornai F (2008). Genetic or pharmacological blockade of norepinephrine synthesis enhances the neurochemical, behavioral, and neurotoxic effects of methamphetamine. J. Neurochem.

[133] Schank JR,  Ventura R,  Puglisi-Allegra S,  Alcaro A,  Cole CD,  Liles LC,  Seeman P,  Weinshenker D (2006). Dopamine beta-hydroxylase knockout mice have alterations in dopamine signaling and are hypersensitive to cocaine. Neuropsychopharmacology.

[134] Pijnenburg AJ,  Honig WM,  Van Rossum JM (1975). Inhibition of D-amphetamine-induced locomotor activity by injection of haloperidol into the nucleus accumbens of the rat. Psychopharmacologia.

[135] Svensson L,  Ahlenius S (1982). Functional importance of nucleus accumbens norepinephrine in the rat. Acta Pharmacol. Toxicol. (Copenhagen).

[136] Ventura R,  Cabib S,  Alcaro A,  Orsini C,  Puglisi-Allegra S (2003). Norepinephrine in the prefrontal cortex is critical for amphetamine-induced reward and mesoaccumbens dopamine release. J. Neurosci.

[137] Volkow ND,  Fowler JS (2000). Addiction, a disease of compulsion and drive: involvement of the orbitrofrontal cortex. Cereb. Cortex.

[138] Paladini CA,  Williams JT (2004). Noradrenergic inhibition of midbrain dopamine neurons. J. Neurosci.

[139] Arencibia-Albite F,  Paladini C,  Williams JT,  Jiménez-Rivera CA (2007). Noradrenergic modulation of the hyperpolarization-activated cation current (Ih) in dopamine neurons of the ventral tegmental area. Neuroscience.

[140] Szot P,  Weinshenker D,  White SS,  Robbins CA,  Rust NC,  Schwartzkroin PA,  Palmiter RD (1999). Norepinephrinedeficient mice have increased susceptibility to seizure-inducing stimuli. J. Neurosci.

[141] Weinshenker D,  Szot P,  Miller NS,  Palmiter RD (2001). Alpha(1) and beta(2) adrenoreceptor agonists inhibit pentylenetetrazole-induced seizures in mice lacking norepinephrine. J. Pharmacol. Exp. Ther.

[142] Giorgi FS,  Ferrucci M,  Lazzeri G,  Pizzanelli C,  Lenzi  
P,  Alessandrì MG,  Murri L,  Fornai F (2003). A damage to locus coeruleus neurons converts sporadic seizures into self-sustaining limbic status epilepticus. Eur. J. Neurosci.

[143] Giorgi FS,  Pizzanelli C,  Biagioni F,  Murri L,  Fornai F (2004). The role of norepinephrine in epilepsy: from the bench to the bedside. Neurosci. Biobehav. Rev.

[144] Hotchkiss AJ,  Gibb JW (1980). Long-term effects of multiple doses of methamphetamine on tryptophan hydroxylase and tyrosine hydroxylase activity in rat brain. J. Pharmacol. Exp. Ther.

[145] Ricaurte GA,  Schuster CR,  Seiden LS (1980). Long-term effects of repeated methylamphetamine administration on dopamine and serotonin neurons in the rat brain: a regional study. Brain Res.

[146] Ricaurte GA,  Guillery RW,  Seiden LS,  Schuster CR,  Moore RY (1982). Dopamine nerve terminal degeneration produced by high doses of methylamphetamine in the rat brain. Brain Res.

[147] Ricaurte GA,  Seiden LS,  Schuster CR (1984). Further evidence that amphetamines produce long-lasting dopamine neurochemical deficits by destroying dopamine nerve fibers. Brain Res.

[148] Lorez H (1981). Fluorescence histochemistry indicates damage of striatal dopamine nerve terminals in rats after multiple doses of methamphetamine. Life Sci.

[149] Green AR,  De Souza RJ,  Williams JL,  Murray TK,  Cross AJ (1992). The neurotoxic effects of methamphetamine on 5-hydroxytryptamine and dopamine in brain: evidence for the protective effect of chlormethiazole. Neuropharmacology.

[150] Kogan FJ,  Nichols WK,  Gibb JW (1976). Influence of methamphetamine on nigral and striatal tyrosine hydroxylase activity and on striatal dopamine levels. Eur. J. Pharmacol.

[151] Bittner SE,  Wagner GC,  Aigner TG,  Seiden LS (1981). Effects of a high-dose treatment of methamphetamine on caudate dopamine and anorexia in rats. Pharmacol. Biochem. Behav.

[152] Eisch AJ, Gaffney M, Weihmuller FB, O’Dell SJ, Marshall JF (1992). Striatal subregions are differentially vulnerable to the neurotoxic effects of methamphetamine. Brain Res.

[153] O’Callaghan JP,  Miller DB (1994). Neurotoxicity profiles of substituted amphetamines in the C57BL/6J mouse. J. Pharmacol. Exp. Ther.

[154] Morgan ME,  Gibb JW (1980). Short-term and long-term effects of methamphetamine on biogenic amine metabolism in extra-striatal dopaminergic nuclei. Neuropharmacology.

[155] Deng X, Ladenheim B, Tsao LI, Cadet JL (1999). Null mutation 
of c-fos causes exacerbation of methamphetamine-induced neurotoxicity. J. Neurosci.

[156] Wagner GC,  Ricaurte GA,  Seiden LS,  Schuster CR,  Miller RJ,  Westley J (1980). Long-lasting depletions of striatal dopamine and loss of dopamine uptake sites following repeated administration of methamphetamine. Brain Res.

[157] Guilarte TR,  Nihei MK,  McGlothan JL,  Howard AS (2003). Methamphetamine-induced deficits of brain monoaminergic neuronal markers: distal axotomy or neuronal plasticity. Neuroscience.

[158] Hirata H,  Ladenheim B,  Carlson E,  Epstein C,  Cadet JL (1996). Autoradiographic evidence for methamphetamine-induced striatal dopaminergic loss in mouse brain: attenuation in CuZn-superoxide dismutase transgenic mice. Brain Res.

[159] Fumagalli F,  Gainetdinov RR,  Wang YM,  Valenzano  
KJ,  Miller GW,  Caron MG (1999). Increased methamphetamine neurotoxicity in heterozygous vesicular monoamine transporter 2 knock-out mice. J. Neurosci.

[160] Gibb JW,  Kogan FJ (1979). Influence of dopamine synthesis on methamphetamine-induced changes in striatal and adrenal tyrosine hydroxylase activity. Naunyn Schmiedebergs Arch. Pharmacol.

[161] Schmidt CJ,  Ritter JK,  Sonsalla PK,  Hanson GR,  
Gibb JW (1985). Role of dopamine in the neurotoxic effects of methamphetamine. J. Pharmacol. Exp. Ther.

[162] Commins DL,  Seiden LS (1986). Alpha-methyltyrosine blocks methylamphetamine-induced degeneration in the rat somatosensory cortex. Brain Res.

[163] Wagner GC,  Walsh SL (1991). Evaluation of the effects of inhibition of monoamine oxidase and senescence on methamphetamine-induced neuronal damage. Int. J. Dev. Neurosci.

[164] Kita T,  Wagner GC,  Philbert MA,  King LA,  Lowndes HE (1995). Effects of pargyline and pyrogallol on the methamphetamine-induced dopamine depletion. Mol. Chem. Neuropathol.

[165] Vergo S,  Johansen JL,  Leist M,  Lotharius J (2007). Vesicular monoamine transporter 2 regulates the sensitivity of rat dopaminergic neurons to disturbed cytosolic dopamine levels. Brain Res.

[166] Guillot TS,  Shepherd KR,  Richardson JR,  Wang MZ,  Li Y,  Emson PC,  Miller GW (2008). Reduced vesicular storage of dopamine exacerbates methamphetamine-induced neurodegeneration and astrogliosis. J. Neurochem.

[167] Marek GJ,  Vosmer G,  Seiden LS (1990). Dopamine uptake inhibitors block long-term neurotoxic effects of methamphetamine upon dopaminergic neurons. Brain Res.

[168] Fumagalli F,  Gainetdinov RR,  Valenzano KJ,  Caron MG (1998). Role of dopamine transporter in methamphetamine-induced neurotoxicity: evidence from mice lacking the transporter. J. Neurosci.

[169] Graham DG,  Tiffany SM,  Bell WR,  Gutknecht WF (1978). Autoxidation versus covalent binding of quinones as the mechanism of toxicity of dopamine, 6-hydroxydopamine, and related compounds toward C1300 neuroblastoma cells *in vitro*. Mol. Pharmacol.

[170] Acikgoz O,  Gonenc S,  Kayatekin BM,  Uysal N,  Pekcetin C,  Semin I,  Gure A (1998). Methamphetamine causes lipid peroxidation and an increase in superoxide dismutase activity in the rat striatum. Brain Res.

[171] Stokes AH,  Hastings TG,  Vrana KE (1999). Cytotoxic and genotoxic potential of dopamine. J. Neurosci. Res.

[172] Sulzer D (2001). Alpha-synuclein and cytosolic dopamine: stabilizing a bad situation. Nat. Med.

[173] LaVoie MJ,  Hastings TG (1999). Dopamine quinone formation and protein modification associated with the striatal neurotoxicity of methamphetamine: evidence against a role for extracellular dopamine. J. Neurosci.

[174] Potashkin JA,  Meredith GE (2006). The role of oxidative stress in 
the dysregulation of gene expression and protein metabolism 
in neurodegenerative disease. Antioxid. Redox Signal.

[175] Battaglia G,  Fornai F,  Busceti CL,  Aloisi G,  Cerrito  
F,  De Blasi A,  Melchiorri D,  Nicoletti F (2002). Selective blockade 
of mGlu5 metabotropic glutamate receptors is protective 
against methamphetamine neurotoxicity. J. Neurosci.

[176] Fornai F,  Bassi L,  Torracca MT,  Scalori V,  Corsini GU (1995). Norepinephrine loss exacerbates methamphetamine-induced striatal dopamine depletion in mice. Eur. J. Pharmacol.

[177] Fornai F,  Giorgi FS,  Alessandrì MG,  Giusiani M,  
Corsini GU (1999). Effects of pretreatment with N-(2-chloroethyl)-N-ethyl-2-bromobenzylamine (DSP-4) on methamphetamine pharmacokinetics and striatal dopamine losses. J. Neurochem.

[178] Ferrucci M, Gesi M, Lenzi P, Soldani P, Ruffoli R, Pellegrini A,  Ruggieri S,  Paparelli A,  Fornai F (2002). Noradrenergic Loss enhances MDMA toxicity and induces ubiquitin-positive striatal whorls. Neurol. Sci.

[179] Kilbourn MR,  Sherman P,  Abbott LC (1998). Reduced MPTP neurotoxicity in the striatum of the mutant mouse tottering. Synapse.

[180] Herkenham M,  Little MD,  Bankiewicz K,  Yang SC,  Markey SP,  Johannessen JN (1991). Selective retention of MPP+ within the monoaminergic systems of the primate brain following MPTP administration: an *in vivo* autoradiographic study. Neuroscience.

[181] Di Chiara G,  Tanda GL,  Frau R,  Carboni E (1992). Heterologous monoamine reuptake: lack of transmitter specificity of neuronspecific carriers. Neurochem. Int.

[182] Antelman SM,  Caggiula AR (1977). Norepinephrine-dopamine interactions and behavior. Science.

[183] Fornai F,  Vaglini F,  Maggio R,  Bonuccelli U,  Corsini GU (1997). Species differences in the role of excitatory amino acids in experimental parkinsonism. Neurosci. Biobehav. Rev.

[184] Kohutnicka M,  Lewandowska E,  Kurzowska-Jastrzebska I,  Czlonkowski A,  Czlonkowska A (1998). Microglial and astrocytic involvement in a murine model of Parkinson’s disease induced by 1-methyl-4- phenyl-1,2,3,6-tetrahydropyridine (MPTP). Immunopharmacology.

[185] Tsuda K,  Tsuda S,  Nishio I,  Masuyama Y,  Goldstein M (1998). Effects of galanin on dopamine release in the central nervous system of normotensive and spontaneously hypertensive rats. Am. J. Hypertens.

[186] Weiss JM,  Bonsall RW,  Demetrikopoulos MK,  Emery MS,  West CH (1998). Galanin: a significant role in depression?. Ann. NY Acad. Sci.

[187] Weiss JM,  Boss-Williams KA,  Moore JP,  Demetrikopoulos MK,  Ritchie JC,  West CH (2005). Testing the hypothesis that locus coeruleus hyperactivity produces depression-related changes *via* galanin. Neuropeptides.

